# Assessing therapeutic decisions in generalized myasthenia gravis: Study protocol

**DOI:** 10.1371/journal.pone.0322168

**Published:** 2025-04-22

**Authors:** Gerardo Gutiérrez-Gutiérrez, Rocío Gómez-Ballesteros, Javier Sotoca, Adrián Ares, Ramón Villaverde, Virginia Reyes, Thaís Armangué, Elisa Salas, Paola Díaz-Abós, Pablo Rebollo, Mónica Sarmiento, Iratxe Escobar, Jorge Maurino, Luis Querol

**Affiliations:** 1 Department of Neurology, Hospital Universitario Infanta Sofía, Madrid, Spain; 2 Medical Department, Roche Farma, Madrid, Spain; 3 Department of Neurology, Hospital Universitario Vall d’Hebron, Barcelona, Spain; 4 Department of Neurology, Complejo Asistencial Universitario León, León, Spain; 5 Department of Neurology, Hospital Universitario Morales Meseguer, Murcia, Spain; 6 Department of Neurology, Hospital Regional Universitario de Málaga, Málaga, Spain; 7 Department of Pediatric Neuroimmunology, Hospital Sant Joan de Déu, Barcelona, Spain; 8 Department of Real World Evidence Studies, IQVIA, Madrid, Spain; 9 Department of Neurology, Hospital de la Santa Creu i Sant Pau, Barcelona, Spain; University of California, Davis, UNITED STATES OF AMERICA

## Abstract

**Background:**

The therapeutic landscape in generalized myasthenia gravis (gMG) has been continuously evolving in recent years, with over five products approved, each with different efficacy, safety, and administration profiles. With the availability of new targeted treatments, physicians are faced with the challenge of therapeutic decision-making tailored to traditional therapeutic goals, individual preferences, and personal experience, seeking optimal disease control with a positive safety profile. In this context of uncertainty and multiple novel choices, this study aims to provide insights into the preferred treatment choices of neurologists managing gMG and to identify demographic, professional or behavioral factors influencing the decision-making process.

**Methods:**

This is a non-interventional, cross-sectional, web-based study involving 150 neurologists treating gMG patients in collaboration with the Spanish Society of Neurology. The primary endpoint will be to assess preferences for different gMG treatment attributes using a conjoint analysis to create hypothetical treatment scenarios. Therapeutic inertia, described as the lack of treatment initiation or intensification when therapeutic goals are not met, will be evaluated as a secondary endpoint through 7 case scenarios simulating real gMG clinical practice situations. Neurologists will also answer a survey composed of demographic, professional, and behavioral characteristics (user resistance behavior, care-related regret, burnout, risk attitude, empathy, work fulfilment, and personality traits) to recognize possible factors influencing decisions.

**Conclusions:**

The study findings will contribute to better understanding of decision-making in gMG under a changing therapeutic landscape with multiple new targeted options, and will identify which factors have a role in affecting those decisions.

## Introduction

Myasthenia gravis (MG) is a rare neurological disorder caused by autoantibodies targeting functionally important molecules in the postsynaptic membrane at the neuromuscular junction, fundamentally disrupting neuromuscular transmission [1]. About 85% of patients present antibodies to the nicotinic acetylcholine receptor (AChR-IgG), whereas a smaller proportion have antibodies to muscle-specific kinase (MuSK-IgG) or lipoprotein-receptor-related protein 4 (LRP4-IgG) [2]. The clinical hallmark of MG is fatigable and debilitating muscle weakness involving facial, bulbar, cervical, axial, and limb muscles [2]. Approximately 85% of patients initially exhibit ocular weakness, presenting as fluctuating ptosis and/or diplopia, as the most frequent initial presentation of MG [2]. Commonly, disease progression to generalized weakness is observed within the first two years from disease onset [2].

Conventional treatment for generalized myasthenia gravis (gMG) has long relied on symptomatic treatment with cholinesterase inhibitors, conventional immunosuppression, immunomodulation with plasma exchange or intravenous administration of high dose immunoglobulin, and possibly thymectomy when indicated [1]. Nevertheless, the gMG therapeutic landscape has rapidly changed over the last few years with the development of multiple targeted immunotherapies such as complement inhibitors and neonatal Fc receptor (FcRn) inhibiting agents [1]. The heterogeneity of these novel therapies and numerous available options, combined with the limited updated evidence regarding management guidelines for gMG, creates a challenge for physicians when making therapeutic decisions [1,3–6]. Furthermore, neurologists’ professional experience, demographic factors or behavioral traits such as resistance to the introduction of innovation, risk attitude, burnout, or regret, could affect the decision-making process, leading to suboptimal decisions and impeding the move from the status quo to patient-tailored therapeutic goals introducing the new options available [7–10].

Considering this changing environment that makes treatment decisions more difficult, this study aims to assess neurologists’ decision-making on gMG treatments and to identify the factors that may be influencing the process.

## Methods

### Study design

**Fig 1:**
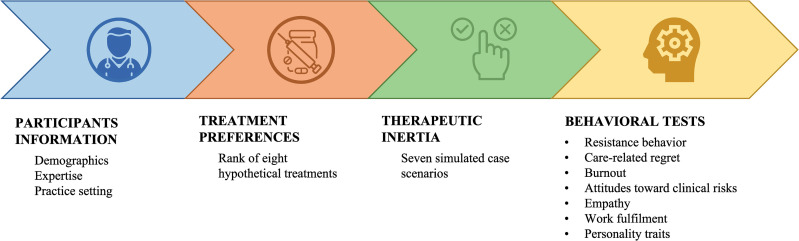
PROMPT-MG study flow.

The PROMPT-MG (SL45180) is a non-interventional, cross-sectional, online study in collaboration with the Spanish Society of Neurology (SEN) to assess therapeutic decisions in AChR-IgG seropositive gMG, covering aspects such as preferences for treatment attributes and therapeutic inertia (TI) (Fig 1). This study will be conducted in accordance with the International Conference on Harmonisation Guidelines for Good Clinical Practice and the ethical principles of the Declaration of Helsinki and was approved by the investigational review board of the Hospital Clínico San Carlos (Madrid, Spain). All participants will provide written informed consent.

### Participants

Neurologists treating gMG patients in Spain will be invited to participate in our study by the SEN. Exclusion criteria will be physicians currently in their residency training period.

### Outcome measures

The primary endpoint of the study will be to assess preferences for different gMG treatment attributes using a conjoint analysis (CA) to create hypothetical treatment scenarios, instead of using the available treatments, as this could lead to potential bias. Conjoint analysis (CA) is a technique based on mathematical psychology and Lancaster’s theory of value. It is designed to discern the significance that individuals place on various attributes of a particular product [11]. This technique involves presenting participants with a sequence of hypothetical product profiles, each defined by a combination of attributes and their respective levels, mirroring the multifaceted decisions physicians encounter in everyday life when choosing between alternatives with diverse attributes [11]. CA is particularly useful for quantifying preferences for non-marketed services and in healthcare, and has been applied successfully to measuring preferences for a diverse range of health applications [12], such as to assess physicians’ preferences in diseases with multiple treatment options like multiple sclerosis [11,13,14].

**Fig 2:**
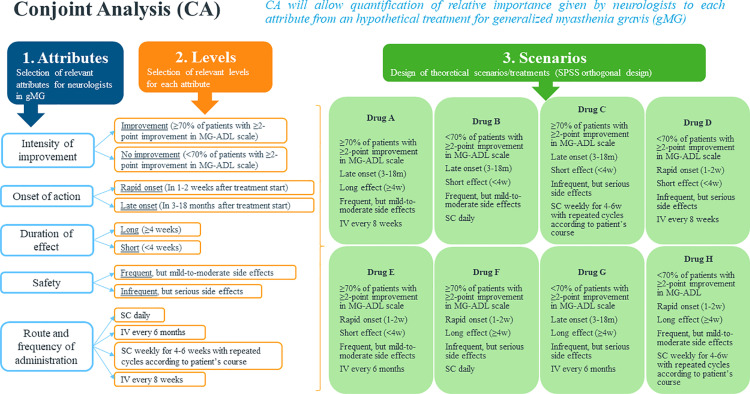
Conjoint Analysis process general overview. IV, intravenous; m, months; MG-ADL, Myasthenia Gravis–specific Activities of Daily Living; SC, subcutaneous; SPSS, Statistical Package for Social Sciences w, weeks.

The CA process starts by selecting relevant attributes for neurologists in gMG (e.g., intensity of patients’ improvement). Once attributes have been chosen, different levels are created to define each attribute. For example, the attribute “intensity of patients’ improvement” will have two levels: improvement [defined as at least 70% of patients reaching an improvement of ≥2 point in Myasthenia Gravis Activities of Daily Living (MG-ADL) scale] and no improvement (defined by the contrary, <70% of patients reaching an improvement of ≥2 point in MG-ADL scale). Once the attributes and their corresponding levels have been defined, scenarios are created by randomization of all levels from all attributes through an orthogonal design using Statistical Package for Social Sciences (SPSS) (Fig 2). Orthogonal design ensures that the effects of each attribute and levels are estimated independently within the set of levels that make up a scenario, and that there is no mixing or correlation between them.

Our CA will evaluate five gMG treatment attributes including intensity of improvement, onset of action, duration of effect, safety risk, and route and frequency of treatment administration, as well as their respective levels (Table 1). All attributes were defined by two levels except route and frequency of administration, which was defined with four levels. They were chosen following a thorough examination of gMG clinical trials and literature reflecting patient perspectives [15–21], and were confirmed in a focus group of seven neurologists with expertise in autoimmune neurological conditions.

**Table 1:** Treatment attributes and levels.

Attributes	Levels
Intensity of improvement	▪ Improvement: ≥70% of patients with ≥2-point improvement in MG-ADL scale ▪ No improvement: <70% of patients with ≥2-point improvement in MG-ADL scale
Onset of action	▪ Rapid onset: In 1–2 weeks after treatment start ▪ Late onset: In 3–18 months after treatment start
Duration of effect	▪ Long: ≥4 weeks **▪** Short: <4 weeks
Safety risk	▪ Frequent, but mild-to-moderate side effects ▪ Infrequent, but serious side effects
Route and frequency of administration	▪ SC daily ▪ IV every 6 months ▪ SC weekly for 4–6 weeks with repeated cycles according to patient’s course ▪ IV every 8 weeks

IV: Intravenous; MG-ADL: Myasthenia Gravis Activities of Daily Living; SC: Subcutaneous.

#### Intensity of improvement.

We will use the MG-ADL scale to describe the levels of this attribute, as it has been reported to be the most common measure to guide treatment decisions in gMG in previous studies [22]. It is an eight-item patient-reported scale developed to assess MG symptoms and their impact on daily activities [23]. The MG-ADL scale scoring system has been used as the primary endpoint of recent clinical trials and it is used in routine clinical management [15–17,19]. Sensitivity and specificity analyses performed with various cut-off points for the change in MG-ADL scale score suggested that a 2-point reduction in the scale indicates a clinically meaningful improvement in MG status [24]. According to clinical trial evidence of new approved targeted treatments, around 70% of patients reach the 2-point reduction in MG-ADL scale [15–17,19]. Thus, this attribute will be divided in two levels: Improvement vs No improvement (Table 1).

#### Onset of action.

Once diagnosis of gMG is confirmed, the aim of management is prompt symptom control and achieving remission or minimal manifestation status as soon as possible [25]. The time to onset of clinical effect of therapies for gMG varies considerably, an aspect that plays a large role, together with the pace and severity of the disease, in choosing the appropriate therapy for a given patient [26]. New approved targeted treatments together with intravenous immunoglobulins (IVIg) have a rapid onset of action (1–2 weeks) [15–17,19,27] compared to immunosuppresants, whose time to reach full effect can vary from 3 months to 18 months [27]. Thus, this attribute will be divided in two levels: Rapid onset vs Late onset. Definitions of these levels can be found on Table 1.

#### Duration of effect.

It is defined as the time from attaining disease control, when patients experience symptom improvement or no symptoms, until they worsen again. Ideally, it should last as long as possible, preventing patients from suffering exacerbations that often require hospitalization, and helping to reduce the disease impact on patients’ quality of life, employment and social life [1]. New anti-FcRn treatments have been reported to achieve their maximum effect at 4 weeks before patients start to have a worsening in their symptoms [15,16]. Thus, this attribute will be divided in two levels: Long vs Short, and the definitions can be found on Table 1.

#### Adverse events (AEs).

AEs are important to patients and represent a barrier to treatment adherence [20,21]. When structuring neurological treatment paradigms among medications with similar efficacy, treatment decisions may be dictated by differences in AEs or treatment burden, among others [28]. Some of these AEs are not severe, but occur frequently over time in a high percentage of patients (e.g., infections, headache, or injection reactions), whereas other therapies are associated with serious but infrequent AEs sometimes resulting from their chronic use in a very low percentage of patients (e.g., progressive leukoencephalopathy, meningococcal infections or neoplasms). Thus, this attribute will be divided in two levels (Table 1).

#### Route and frequency of treatment administration.

The various options for route and frequency of administration of treatments for gMG can influence the preferences of choice during therapeutic management. Some studies suggest a possible interaction between dose frequency and route of administration [29]. Thus, we combined both characteristics into a single attribute with four levels (Table 1).

To prevent cognitive fatigue while completing the survey due to having too many choices, only five attributes were included. The inclusion of more attributes/levels would have exponentially increased the number of hypothetical treatments, thus turning a simple exercise into a complicated one. However, though not included, further attributes were considered throughout the process, including: availability of pregnancy data (possible levels: unknown or high risk during pregnancy vs low risk during pregnancy), mechanism of action (possible levels: stopping damage at the end vs acting at the beginning of the process), availability of real-world data (possible levels: available data vs no available data), elimination of auto-antibodies (yes vs no), and treatment administration/burden for patients (including time of administration, number of hospital visits…). These attributes were discarded in the process to leave the five main attributes described above (intensity of improvement, onset of action, duration of effect, adverse events, and route and frequency of treatment administration).

**Fig 3:**
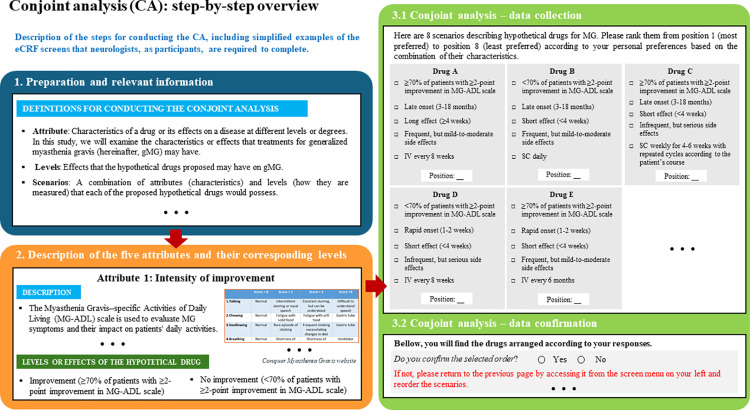
Step-by-Step Overview of completing the Conjoint Analysis (CA) in the eCRF. It is a simplified representation of the eCRF screen sequence that neurologist participants must follow and complete, guided by the specific instructions displayed on each screen. The ellipsis symbol (…) indicates that the actual eCRF screens contain additional information or content not shown here. CA, conjoint analysis; eCRF, electronic Case Report Form; IV, intravenous; MG, myasthenia gravis; MG-ADL, Myasthenia Gravis–specific Activities of Daily Living scale; SC, subcutaneous.

In the end, to assess preferences for different gMG treatment attributes, eight hypothetical treatment scenarios with unique attribute and level combinations were created (Table 2, options A to H). Participating neurologists will have to rank them from most preferred to least preferred, and attribute preferences will be calculated (Fig 3).

**Table 2:** Set of treatment scenarios.

Treatment scenarios	Improvement of symptoms	Onset of action	Duration of effect	Safety	Route and frequency of administration
A	Improvement	Late onset	Long	Frequent, but mild-to-moderate side effects	IV every 8 weeks
B	No improvement	Late onset	Short	Frequent, but mild-to-moderate side effects	SC daily
C	Improvement	Late onset	Short	Infrequent, but serious side effects	SC weekly for 4–6 weeks with repeated cycles according to patient’s course
D	No improvement	Rapid onset	Short	Infrequent, but serious side effects	IV every 8 weeks
E	Improvement	Rapid onset	Short	Frequent, but mild-to-moderate side effects	IV every 6 months
F	Improvement	Rapid onset	Long	Infrequent, but serious side effects	SC daily
G	No improvement	Late onset	Long	Infrequent, but serious side effects	IV every 6 months
H	No improvement	Rapid onset	Long	Frequent, but mild-to-moderate side effects	SC weekly for 4–6 weeks with repeated cycles according to patient’s course

IV: Intravenous; SC: Subcutaneous. Complete levels are described in Table 1: Improvement: ≥70% of patients with ≥2-point improvement in MG-ADL scale; No improvement: <70% of patients with ≥2-point improvement in MG-ADL scale; Rapid onset: in 1–2 weeks after treatment start; Late onset: in 3–18 months after treatment start; Long: ≥4 weeks; Short: <4 weeks.

As a secondary endpoint, we will explore the prevalence of TI in gMG through 7 case scenarios simulating real AChR-IgG seropositive gMG clinical practice situations designed by the research team (S1 Supplementary Material). TI is classically defined as the lack of treatment initiation or intensification when therapeutic goals are not met [30], but it can be further defined depending on the context of the disease. For example, in neuromyelitis optica spectrum disorder, it has been defined as not initiating a high-efficacy treatment or not escalating to a high-efficacy treatment when therapeutic goals are not met due to the devastating nature of relapses in the disease [31], and in multiple sclerosis it has been defined as not incorporating new biomarkers in clinical decisions [32]. To assess TI in this study, we first described what a suboptimal response in gMG would be: “a MG-ADL scale score of at least 1 after enough time receiving the treatment and/or the presence of severe/serious adverse events associated with the treatment”, thereby ensuring a demanding therapeutic goal approach defined as “a MG-ADL scale score of 0 with a possible minor weakness on examination of some muscles” [33–35]. For the assessment of suboptimal responses, new targeted treatments will be considered as more efficacious or with a greater probability of achieving the aforementioned goal, and with a better benefit-risk profile than off-label treatments due to their solid results in clinical trials and the subsequent experience gained [15–19]. Corticosteroids, azathioprine and mycophenolate mofetil will be classified together as a group of lower efficacy, with the aim of pursuing the best possible outcomes if a patient shows worsening of symptoms, reflecting no tolerance to weakness. Cases included were thoughtfully constructed to assess if the location of the weakness experienced (extremities vs bulbar), the therapy used (monotherapy vs combination) or a rapid progression of symptoms would lead to different therapeutic strategies, and none of the core cases was structured to lead to a confusing situation (e.g., only presence of occasional ptosis).

Four additional case scenarios will be included for exploratory and descriptive purposes, including an ocular form of MG, a MuSK-IgG form of MG, discomfort from treatment side effects reported by the patient, and patients with a past myasthenic crisis (S1 Supplementary Material, cases 9–12). Furthermore, we will assess herding phenomenon, i.e., the phenomenon by which individuals follow the behavior of others rather than deciding independently on the basis of their own private information, through a case scenario as an additional exploratory endpoint (S1 Supplementary Material, case 13) [36]. To avoid random answers from participants, a control case scenario will be included to remove such profiles from the sample (S1 Supplementary Material, case 8).

Demographic and professional characteristics will be collected to identify the potential association with therapeutic decisions (Table 3). To further explore which factors could potentially have an impact on decision-making, we will use different questionnaires, including the assessment of resistance behavior (adapted to the introduction of new targeted therapies for gMG from the user resistance model of Kim and Kankanhalli) [37,38], care-related regret (Regret Intensity Scale, RIS-10) [39], burnout (single-item emotional exhaustion) [40], attitudes towards clinical risks [10], empathy (Jefferson Scale of Physician Empathy, JSPE) [41], work fulfillment (Utrecht Work Engagement Scale, UWES) [42], and personality traits (Big Five Inventory, BFI-10) [43]. A summary of these assessments and scores can be found in Table 4. Additionally, to further explore how decisions are made in the gMG clinical setting, we will include questions about measures used to follow patients’ evolution and the importance given to the mechanism of action of treatments used for the disease.

**Table 3:** Demographic characteristics, professional background, and practice setting.

Characteristics
Age
Gender
Years of experience as neurologist
Years of experience managing MG patients
Degree of specialization: neuromuscular specialist, demyelinating diseases specialist, general neurologist involved in the care of MG patients
Practice setting: academic hospital (yes/no)
MG-specific consultation: yes/no
Number of MG patients managed by the neurologist in one month
Number of neurologists who manage MG patients with the participant neurologist
Participation in MG clinical trials: yes/no
Co-authorship of peer-reviewed publications in the last 3 years: yes/no
Attended neuromuscular congresses in the last year: yes/no
Sick leave in the last year and for how long: yes/no and total number of days

MG: Myasthenia Gravis.

**Table 4:** Outcome measures.

Outcome	Measure	Scoring and interpretation	Range
**User resistance behavior**	User resistance model	A 34-item questionnaire to assess user resistance behavior covering different aspects (resistance behavior, resistance to change, perceived usefulness, perceived ease of use, perceived value, colleagues’ opinions, self-efficacy for change, and organizational support for change). It has been adapted to the introduction of new targeted treatments in gMG clinical practice. Each item is rated on a 7-point Likert scale ranging from 1 (strongly disagree) to 7 (strongly agree). Higher total scores indicate greater resistance to change.	1-7
**Care-related regret**	RIS-10	A 10-item instrument to assess regret caused by a past care-related event, covering affective, physical, and cognitive aspects. Each item is assessed on a 5-point Likert scale from 1 (not at all) to 5 (to a very great extent). Higher scores indicate a higher intensity of regret, with scores of ≥3 indicating a moderate-to-high intensity of regret.	1-5
**Burnout**	Single-item emotional exhaustion	A single item to assess burnout as: *Overall, based on your definition of burnout, how would you rate your level of burnout?* Score ranges from 1 (no symptoms) to 5 (completely burned out), with scores ≥3 considered positive for burnout.	1-5
**Risk attitude**	Single-item clinical risk attitude	A single item to measure the clinical risk when adopting a new drug: *How likely are you to engage in clinical risks (e.g., recommending a treatment which is new to your usual practice or is controversial)?* Score ranges from 1 (very unlikely) to 5 (very likely), with scores of 1 indicating risk aversion and scores of ≥3 indicating a risk-taker profile.	1-5
**Empathy**	JSPE	A 20-item scale to measure empathy in physicians. Each item is scored on a 7-point Likert scale from 1 (strongly disagree) to 7 (strongly agree). Higher scores indicate a greater degree of empathy.	20-140
**Work fulfilment**	UWES	A 9-item scale to measure work engagement, covered by vigor, dedication, and absorption dimensions. Each item is scored from 0 (never) to 6 (always). Higher scores indicate greater work fulfilment.	0-6
**Personality traits**	BFI-10	A 10-item scale to assess the Big Five personality traits (Extraversion, Agreeableness, Conscientiousness, Emotional Stability/Neuroticism, and Openness to experience). Each item score ranges from 1 (strongly disagree) to 5 (strongly agree).	1-5

BFI-10: Big Five Inventory; JSPE: Jefferson Scale of Physician Empathy; RIS-10: Regret Intensity Scale; UWES: Utrecht Work Engagement Scale.

#### User resistance behavior.

Resistance is defined as the informal and covert behavior of an individual in response to a perceived or actual threat in an attempt to maintain the status quo, and has historically been viewed with negative consequences due to its potential impact on organizational success [7]. User resistance behavior has been described to be affected either directly or indirectly by different aspects, including resistance to change, perceived usefulness, perceived ease of use, perceived value of the innovation, colleagues’ opinions, self-efficacy for change, and organizational support for change [38]. We will assess the user resistance behavior to abandoning the actual status quo in gMG with the introduction of new targeted treatments in clinical practice by modifying the user resistance model of Kim and Kankanhalli in this respect [37,38]. We will use a 34-item questionnaire covering all aspects that affect resistance behavior, with items rated from 1 (strongly disagree) to 7 (strongly agree).

#### Care-related regret.

Regret is an emotion that occurs after an experience in which one feels responsible for negative outcomes or thinks that a different choice would have led to a better outcome [39]. The experience of regret related to medical decisions made by healthcare professionals can have an impact on their health and quality of life, as well as on their practice [9]. The RIS-10 will assess the presence of care-related regret and its intensity regarding a past medical decision with a patient, covering emotional, physical, and cognitive aspects of regret [39]. Participants will be asked to rate their agreement with each item on “how they feel now about that experience” from 1 (not at all) to 5 (to a very great extent), with higher scores indicating higher regret intensity.

#### Burnout.

Professional burnout is a condition resulting from chronic workplace stress that has not been successfully managed. It negatively influences physicians’ physical and emotional health and has an impact on therapeutic decisions [8,9,44]. We will use the non-proprietary single-item burnout measure to assess occupational burnout. It asks respondents to rate their burnout level based on different definitions using a 5-point scale [40].

#### Attitudes toward clinical risks.

Risk attitude is considered a fundamental part of decision-making under uncertainty and is hypothesized to affect medical decisions [10,45]. As an individual’s level of risk taking is relatively inconsistent across situations depending on the circumstances, we will specifically assess clinical risks when adopting new drugs [10]. Physicians will be asked directly about their clinical risk taking when recommending a new or controversial treatment in clinical practice, scored on a five-point Likert scale from 1 (very unlikely) to 5 (very likely) [10].

#### Empathy.

Physicians’ empathy is the ability to understand the patient’s situation, perspective, and feelings [41,46]. It comprises cognitive, affective, and behavioral dimensions, and involves knowing how to transmit this understanding to patients in a supportive manner to prevent or alleviate their suffering [41,46]. It may be affected by factors such as workload, burnout, emotional overload and lack of good mentors, and it might affect decision-making [46]. To assess the degree of empathy of participating neurologists, we will use the JSPE, a 20-item scale with higher scores indicating a greater degree of empathy [41].

#### Work fulfilment.

Work engagement is the assumed opposite of burnout, a positive work-related state of fulfilment that is characterized by vigor, dedication, and absorption [42]. Contrary to those who suffer from burnout, engaged employees have a sense of energetic and effective connection with their work activities and they see themselves as able to deal well with the demands of their job [42]. We will use the UWES to measure work engagement, a 9-item scale grouped into three subscales that reflect the underlying dimensions of engagement. All items are scored from 0 (never) to 6 (always) [42].

#### Personality traits.

Physicians’ personality has been reported to influence decision-making and professionals’ well-being, and has been associated with the use of more intensive therapy or worse psychological health under uncertainty or stressful situations [47]. The BFI-10 is a short questionnaire for conceptualization of human personality based on five relatively independent factors (extraversion, agreeableness, conscientiousness, neuroticism, and openness to experience) that account for phenotypic personality variations between people [43]. Each item score ranges from 1 (strongly disagree) to 5 (strongly agree) [43].

## Data Management

Data from neurologists will be recorded in a database specifically designed for this study via an online survey. Neurologists will provide their written informed consent and confirm their eligibility for the study electronically. Subsequently, they will complete the information about their demographic characteristics, professional background, and behavioral assessments, and will perform both the attribute preferences and TI assessment exercises. Regular reviews of the database will be conducted to identify and address any potential inconsistency, ensuring its resolution. Upon reaching the proposed sample size for the study outcomes, the study database will be locked, and the statistical analyses will be conducted.

### Statistical considerations and sample size

It is estimated that approximately 150 neurologists in Spain will participate. Our sample size calculation was estimated based on the ideal number of subjects needed to conduct the preference exercise. Considering that eight theoretical scenarios will be presented to the participating neurologists, and that a regression model will be constructed with their responses, requiring a minimum of 15 observations per scenario, a total of 120 neurologists would be needed for the exercise. The general rule of having at least 15 observations is used to ensure that the model has sufficient statistical power to obtain reliable estimates and to avoid overfitting problems [48]. As it is not uncommon for some participants to not complete the entire questionnaire or exhibit reversals (subjects whose preferences show the opposite relationship to what is expected), an additional 30 subjects will be included in the sample. In Spain, there are around 1,600 neurologists. Of those, 700 could potentially treat patients with neuroimmune diseases (central nervous system or peripheral). Then, this sample represents approximately 9.3% of the total population of neurologists and a 21.4% of the population of neurologists who could treat patients with neuroimmune diseases.

To create the hypothetical treatment scenarios, we applied an orthogonal design with the SPSS.

Descriptive statistics will be presented for all variables. Measures of central tendency (mean and median), variability/dispersion (standard deviation and interquartile ranges) will be used for continuous variables, whereas categorical variables will be presented with distributions of absolute and relative frequencies (percentages of groups).

An ordinary least squares regression model will be used to estimate parameters, and results will be presented in terms of utilities and relative and individual importance assigned to each attribute. These will be obtained by dividing the importance of a factor (maximum difference in utility values assigned to the levels) by the sum of all individual importance scores.

Pearson’s R and Kendall’s τ coefficients will be used to provide measures of the correlation between observed and estimated preferences to assess the model’s goodness of fit. Individual importance of each attribute (or level) for obtaining information about factors related to the importance assigned to different attributes will be explored according to participants´ characteristics using bivariate tests. We will use STATA 17 (College Station, TX: StataCorp LP) to conduct the analyses.

### Study status and timeline

The study status in ongoing. Participant recruitment and data collection began in April 12, 2024. The expected date for database completion is August 2024.

## Discussion

The PROMPT-MG is a non-interventional, cross-sectional, web-based study to assess the decision-making process after the introduction of novel targeted treatments in gMG. Our study will assess treatment preferences and therapeutic inertia among neurologists treating gMG patients and which demographic, professional, or behavioral factors could have an influence on these decisions.

The therapeutic landscape in gMG has evolved in recent years, with over five products (eculizumab, efgartigimod, ravulizumab, zilucoplan, and rozanolixizumab) approved, each with different efficacy, safety, and administration profiles. Unlike off-label immunosuppressants, all these new treatments have a selective target conferring a probably better long-term efficacy and safety profile. Eculizumab, ravulizumab, and zilucoplan block the complement cascade by binding to C5 [17,19,49,50], whereas efgartigimod and rozanolixizumab prevent the recycling of autoantibodies through the FcRn receptor pathway [15,16,51]. New treatments are divided in these two modes of action, but factors such as efficacy and safety profile, onset of action, clinical practice experience, physicians’ perception, pregnancy data, and different administration profiles could have an impact on preferences for one of these treatments [11,13,14]. However, there is limited information available on how current therapeutic choices are made in gMG [52]. A recent survey exploring physicians’ decisions on the use of novel therapeutic agents was conducted in the United States including 81 neuromuscular specialists [52]. They found that azathioprine and mycophenolate mofetil were the treatments most frequently used as first-line, leaving rituximab for refractory cases, and using FcRn and complement inhibitors as bridge therapies [52]. Cost and lack of experience were the main disadvantages for the use of these new therapies, whereas affordability and safety profile were the most important attributes to differentiate between novel treatments in their choice as first line [52].

With the approval of these treatments, new and more demanding therapeutic goals should be defined for patients so they may benefit from the right therapy early in their disease, thus preserving their quality of life and preventing the future constant symptoms that have been reported [3,5,20,53–57]. A change in the therapeutic paradigm has been seen in other neuroimmune diseases, such as multiple sclerosis or neuromyelitis optica spectrum disorder, where approval of new treatments led to a concept known as flipping the pyramid or using high-efficacy targeted treatments early in the disease – instead of a watchful wait between lower efficacy treatments before escalating – to preserve the neuronal reserve in the long term [31,58–60]. A similar approach could be extrapolated to gMG, implementing a rapid escalation due to demanding goals with the aim of preserving the post-synaptic membrane connections at the neuromuscular junction, avoiding the patient cycling between various off-label low efficacy treatments [54,61]. In our study, we defined that a suboptimal response would be an MG-ADL scale score of at least 1 point after enough time receiving the treatment and/or the presence of severe/serious adverse events associated with the treatment [33,35], and thus, not switching to a higher efficacy treatment would imply the presence of therapeutic inertia.

Previous studies have found an influence between physicians’ demographic, professional, and/or behavioral characteristics and therapeutic decisions in neuroimmune conditions. Factors such as years of experience, disease specialization, number of patients managed, type of hospital, comfort with medical decision-making, poor empathy, care-related regret, risk attitude, or prioritizing patient safety/tolerability in treatment selection have been associated with therapeutic inertia, treatment preferences or the presence of herding phenomenon [30,31,36,47,62]. We will assess these aspects including resistance behavior to the introduction of novel therapies, work fulfilment, or personality traits to see the impact on decision-making in the current context of gMG.

Several limitations should be noted regarding our study protocol. First, the cross-sectional design will not allow us to assess changes or causal relationships over time in decision-making, but it is a starting point to assess the current picture. Second, attributes included in the preferences’ exercise are limited to only five and some may have been left out. The same situation can be applied to the number of levels defined for each attribute. The “onset of action” attribute is defined by only two levels, one of them covering a broad period of time (3–18 months) that could imply a bias in the results. Furthermore, some levels could have benefited from including additional details, such as the percentage of frequent/infrequent side effects. Nevertheless, including excessive information per level or increasing the number of attributes/levels would lead to numerous scenarios, which could make the decision-making process cognitively fatiguing. Third, rituximab and IVIg were not included as possible responses in core clinical cases. However, increasing the options would have led to cognitive fatigue as stated before, and IVIg and rituximab are not usually used as long-term therapies for AChR seropositive gMG patients in Spain, unless failure has occurred with the other treatment options. Despite these limitations, our study will include different exercises for assessing decisions on gMG covering multiple aspects that could have an impact on the outcome.

In conclusion, our findings will offer valuable insights into how decisions are made in gMG and factors influencing these decisions, providing complementary information that may be useful to update treatment guidelines, design policy decisions, and ensure specific medical educational interventions to achieve the best care for gMG patients.

## Supporting information

S1 Supplementary Material Simulated case scenarios as presented to participants. (DOCX)
