# Supplementary material for: Assessing therapeutic decisions in generalized myasthenia gravis: Study protocol
Source: PLoS One. 2025 Apr 22;20(4):e0322168. doi: 10.1371/journal.pone.0322168 (PMC12013924; doi:10.1371/journal.pone.0322168)
Supplement: S1 Supplementary Material — (DOCX) [file pone.0322168.s001.docx]

*Study Protocol*

**Assessing Therapeutic Decisions in generalized Myasthenia Gravis: Research Protocol**

**Supplementary Material**

Simulated case scenarios as presented to participants. Cases 1-7 will be used to assess therapeutic inertia. The rest of the cases will be descriptive and exploratory except for case 8, which will be used as a control case to avoid random answers (profiles who answer bold options in case 8 will be removed from the sample) and case 13 to assess herding phenomenon (answer in bold will be considered as herding presence):

**Please answer the following case scenarios as in a hypothetical situation of equivalent conditions of access for all drugs, without the decision to treat with one drug or another being determined by usage protocols, guidelines or its price, or by the particular conditions of the patient, such as being a woman of fertile age with pregnancy desire or other additional situations that may influence decision-making. Furthermore, the use of pyridostigmine or performance of a thymectomy in the past will not be considered or included in the possible responses. In the selection of the drug only a single option will be considered, although combinations may occur.**

**Case 1:** A 45-year-old woman diagnosed with seropositive antibodies against the nicotinic acetylcholine receptor (AChR-Abs+) gMG for 2 years and on treatment with azathioprine and corticosteroids for a year. She comes to the consultation with constant drooping of the eyelid and reporting diplopia when driving. She also reports having difficulty washing her hair in the shower, getting up from a chair, and some episodes of fluid loss from her nose when drinking water, findings that are confirmed by the Quantitative Myasthenia Gravis scale (QMG). No confounding factors were found that could have altered the patient's clinical status.

What would be your treatment strategy?

a) Maintain treatment and reassess the patient in 3 months

b) Ravulizumab

c) Mycophenolate mofetil

d) Efgartigimod

e) Increase the dose of corticosteroids

f) Rozanolixizumab

g) Zilucoplan

**Case 2:** A 38-year-old woman diagnosed with gMG (AChR-Ab+) 2 years ago for weakness in the upper extremities and diplopia. When treatment with corticosteroids and azathioprine was prescribed, an improvement in double vision was observed, but in the last consultation she reported that she still had to take breaks when combing her hair every morning, among other activities, confirming proximal weakness in the QMG. No confounding factors were found that could have altered the patient's clinical status.

What would be your treatment strategy?

a) Rozanolixizumab

b) Zilucoplan

c) Mycophenolate mofetil

d) Efgartigimod

e) Ravulizumab

f) Maintain treatment and reassess the patient in 3 months

g) Increase the dose of corticosteroids

**Case 3**: A 27-year-old woman presents with muscle weakness affecting her extremities, constant ptosis, intermittent diplopia and bulbar symptoms that have led her to change her diet to a soft food diet, but she still has difficulty swallowing and it causes her to choke frequently. She underwent cycles of plasmapheresis and was diagnosed with gMG with AChR antibodies.

What would be your treatment strategy?

a) Zilucoplan

b) Corticosteroids

c) Efgartigimod

d) Mycophenolate mofetil

e) Combination of classic immunosuppressants

f) Ravulizumab

g) Azathioprine

h) Rozanolixizumab

**Case 4**: A 57-year-old man diagnosed with gMG (AChR-Ab+) has been treated with azathioprine for 3 years without any symptoms since then. The patient comes to the consultation and comments that he has to take rest periods when doing daily tasks such as washing his hair, confirming weakness in the upper limbs in the QMG. No confounding factors were found that could have altered the patient's clinical status.

What would be your treatment strategy?

a) Efgartigimod

b) Ravulizumab

c) Mycophenolate mofetil

d) Rozanolixizumab

e) Maintain azathioprine and reassess the patient in 3 months

f) Add corticosteroids

g) Zilucoplan

**Case 5**: A 56-year-old man diagnosed with gMG (AChR-Ab+) has been treated with azathioprine for 4 years without any symptoms since then. The patient comes to the consultation with his companion, who comments that in recent months there have been times when he has nasal speech and sometimes it is difficult for him to pronounce words correctly, confirming oropharyngeal bulbar involvement in the QMG. No confounding factors were found that could have altered the patient's clinical status.

What would be your treatment strategy?

a) Ravulizumab

b) Zilucoplan

c) Mycophenolate mofetil

d) Efgartigimod

e) Maintain azathioprine and reassess the patient in 3 months

f) Rozanolixizumab

g) Add corticosteroids

**Case 6:** A 60-year-old man diagnosed with gMG (AChR-Ab+) has been treated with corticosteroids and azathioprine for 1 year without any symptoms. The patient comes to the consultation reporting that he has difficulty swallowing solids and feels much more comfortable with soft/liquid foods, confirming oropharyngeal bulbar involvement by the QMG. No confounding factors were found that could have altered the patient's clinical status.

What would be your treatment strategy?

a) Mycophenolate mofetil

b) Efgartigimod

c) Rozanolixizumab

d) Increase the dose of corticosteroids

e) Ravulizumab

f) Maintain treatment and reassess the patient in 3 months

g) Zilucoplan

**Case 7:** 46-year-old woman diagnosed 7 years ago with gMG (AChR-Ab+). She presented a myasthenic crisis under treatment with corticosteroids 3 years ago, which was resolved with several cycles of intravenous immunoglobulins, and was then prescribed mycophenolate mofetil, a treatment which she continued on. However, in recent years she has had recurrent ptosis and diplopia, which worsen at the end of the day, affecting her quality of life. No confounding factors were found that could have altered the patient's clinical status.

What would be your treatment strategy?

a) Ravulizumab

b) Zilucoplan

c) Azathioprine

d) Efgartigimod

e) Maintain mycophenolate mofetil and reassess in 4 months

f) Rozanolixizumab

g) Add corticosteroids

**Case 8:** A 63-year-old man diagnosed with gMG (AChR-Ab+) treated with ravulizumab. At the follow-up visit he reports feeling well and there are no detectable symptoms.

What would be your treatment strategy?

**a) Corticosteroids**

**b) Azathioprine**

**c) Mycophenolate mofetil**

**d) Combination of classic immunosuppressants**

e) Gradually withdraw treatment

f) Maintain treatment and reassess the patient in 6 months

**g) Efgartigimod**

**h) Rozanolixizumab**

**i) Zilucoplan**

**Case 9 (longitudinal):** A 59-year-old woman who has had eyelid drooping and double vision for the past 9 months when watching television and reading books. In the neurological examination, she presented moderate bilateral ptosis that improved with the ice test and high titers of AChR antibodies, diagnosing her with ocular MG and starting corticosteroids. After 5 months, the patient returned to the consultation, reporting a slight objective improvement. She did not present ptosis, but she did comment that double vision persisted from time to time and that shampoo enters in her eyes when taking a shower because she cannot close them completely. No confounding factors were found that could have altered the patient's clinical status.

What would be your treatment strategy?

a) Maintain corticosteroids and reassess the patient in 3 months

b) Increase the dose of corticosteroids

b) Azathioprine

d) Mycophenolate mofetil

e) Efgartigimod

f) Ravulizumab

g) Rozanolixizumab

h) Zilucoplan

The dose of corticosteroids is increased and three months later the patient returns with bilateral ptosis and constant diplopia, something very limiting for her at work and in her quality of life. No confounding factors were found that could have altered the patient's clinical status.

What would be your treatment strategy?

a) Maintain corticosteroids and reassess the patient in 3 months

b) Efgartigimod

c) Azathioprine

d) Mycophenolate mofetil

e) Combination of classic immunosuppressants

f) Increase again the dose of corticosteroids

g) Ravulizumab

h) Rozanolixizumab

i) Zilucoplan

**Case 10:** 30-year-old woman diagnosed with gMG (AChR-Ab+) 5 years ago, treated with methotrexate and low-dose corticosteroids and without symptoms of weakness/fatigue since then. In her last visit, she reports being fed up with gaining weight and the “moon face,” despite the nausea and vomiting she has from the treatment and the constant visits to specialists and the burden it places on her life.

What would be your treatment strategy?

a) Maintain corticosteroids and methotrexate and reassess the patient in 3 months

b) Ravulizumab

c) Eliminate methotrexate

d) Rozanolixizumab

e) Mycophenolate mofetil

f) Efgartigimod

g) Eliminate corticosteroids

h) Azathioprine

i) Zilucoplan

**Case 11:** A 35-year-old woman presenting with upper extremity weakness, dysphagia and dysarthria, with high muscle-specific kinase (MuSK) antibody titers, was diagnosed with gMG.

What would be your treatment strategy?

a) Corticosteroids

b) Rozanolixizumab

c) Combination of classic immunosuppressants

d) Mycophenolate mofetil

e) Rituximab

f) Azathioprine

**Case 12:** 65-year-old man diagnosed with gMG (AChR-Ab+) 5 years ago. He started treatment with corticosteroids and azathioprine, and when the dose of corticosteroids was reduced, he had a myasthenic crisis 2 years ago, which was treated with cycles of plasmapheresis, and corticosteroids were again included in the treatment with azathioprine until now. He recently called the office reporting pain in the neck, observing axial weakness in the consultation when asking for head elevation in the supine position. No confounding factors were found that could have altered the patient's clinical status.

What would be your treatment strategy?

a) Ravulizumab

b) Rozanolixizumab

c) Mycophenolate mofetil

d) Efgartigimod

e) Maintain treatment and reassess the patient in 3 months

f) Increase the dose of corticosteroids

g) Zilucoplan

**Case 13:** A 42-year-old woman diagnosed with gMG for 3 years [Myasthenia Gravis Foundation of America (MGFA) IIa] was treated with pyridostigmine and azathioprine without significant adverse events. Three months ago, she developed increased weakness in her legs that made very difficult for her to get up from bed or a chair without the help of her hands or others and which she resolved spontaneously in 2-3 weeks.

Her neurological exam has not changed since her last evaluation last year [Myasthenia Gravis Activities of Daily Living (MG-ADL) score of 0]. Normal blood test. She is not taking any new medications. The patient and her husband expressed some concerns about her recent symptoms and decided to seek a second opinion. She was evaluated by a neurologist with experience in neuromuscular disorders from a university hospital in the city of Madrid who recommended changing treatment to ravulizumab.

The patient returns to your consultation. What would be your treatment strategy?

a) Maintain azathioprine and reassess the patient in 3 months

**b) Start ravulizumab as recommended by your neuromuscular colleague.**

c) Change to mycophenolate mofetil.

d) Maintain azathioprine, order new tests and reassess the patient in 6 months.
